# Estimation of the Nutrient and Chlorophyll a Reference Conditions in Taihu Lake Based on A New Method with Extreme–Markov Theory

**DOI:** 10.3390/ijerph15112372

**Published:** 2018-10-26

**Authors:** Liang Wang, Yulin Wang, Haomiao Cheng, Jilin Cheng

**Affiliations:** 1 School of Hydraulic Energy and Power Engineering, Yangzhou University, Yangzhou 225009, China; right628@126.com; 2 School of Environmental Science and Engineering, Yangzhou University, Yangzhou 225127, China; yzchhm@yzu.edu.cn; 3 Key Laboratory of Integrated Regulation and Resource Development on Shallow Lake of Ministry of Education, College of Environment, Hohai University, Nanjing 210098, China

**Keywords:** reference condition, Markov chain, extreme method, shallow lake, high precision

## Abstract

The nutrient reference conditions of lakes play a key role for lake water quality control and water resource management. The inferential models are important methods for calculating reference values; however, the dependence and “cluster” in time series make time series data difficult to be applied in these methods. A new method based on Markov chain theory, which is used for modeling the dependence of data, and extreme statistics was proposed. The new method was used to estimate the nutrient and chlorophyll a reference conditions in Taihu Lake, which is the third largest freshwater lake in China. The results showed that there was remarkable dependence between the effective observations of total nitrogen (TN), total phosphorus (TP), and chlorophyll a. The recommended reference conditions of TN, TP, and chlorophyll a in Taihu Lake were 0.69 mg/L, 0.029 mg/L, and 1.89 μg/L. Their 95% confidence intervals were 0.62–0.76 mg/L, 0.028–0.030 mg/L, and 1.55–2.23 μg/L. These results were consistent with previous researches, which showed that the proposed method is reliable and effective. The length of the intervals was remarkably reduced when compared with several methods. This implied that the proposed method could make full use of the observation data in time series and significantly improve the precision of the estimation results of reference conditions. In general, the proposed method could provide high precision and reliable lake nutrient reference conditions, which would be beneficial to lake water resource management and can be used for estimating the TN, TP, and chlorophyll a reference conditions of other lakes.

## 1. Introduction

Lake water resource management is becoming more and more important due to increasingly serious water pollution problems, especially in the development region of eastern China. Estimation of the lake nutrient reference conditions plays a key role in pollution control and water resource management and provides a baseline for developing water quality benchmarks [1]. Therefore, it is necessary to study the nutrient reference conditions of lakes.

Lake nutrient reference conditions are considered as the nutrient levels least impacted by human activities in lakes [1,2,3]. The total phosphorus (TP), total nitrogen (TN), chlorophyll a, and Secchi depth (SD), which are frequently used to represent the trophic condition in lakes [4,5], are often chosen as representative variables of nutrient reference conditions in lakes [1,3]. In undeveloped areas, the reference conditions can be worked out by analyzing the monitoring data of typical environmental variables of “reference lakes” [3]. However, it is difficult to find “reference lakes” in developed industrial or agricultural regions, so the nutrient reference conditions should be considered as the best attainable nutrient conditions in these areas [1,6].

Many approaches have been applied to recommend reference conditions in the regions affected by industrial and agricultural activities. Generally speaking, the methods can be divided into three categories [7]: historical data analysis, inferential models, and processed-based models. The processed-based model methods often employ the watershed model and dynamic system theory [7,8] and require less data than the other two approaches. However, these models need a deep understanding of the impacts of human activities on the biogeochemical processes in the lake. Historical data analysis and inferential models involve the application of statistical methods and require more observations, especially for historical data analysis, but these methods have fewer requirements for understanding the biogeochemical processes in lakes. In practice, both historical data analysis and inferential models have been applied frequently for the full use of the existing observation data.

Usually, historical data analysis methods are based on single variable descriptive statistics and quantile selection. Nutrients and chlorophyll a observations are collected and analyzed. Then, the quantiles selected directly from observations are signed as reference conditions. The lake population distribution approach recommended by the U.S. EPA [1,2] and EU [3], the trisection approach used in Kansas [9] and Chao Lake [10], and the frequency analysis used in Taihu Lake [11] are typical application cases of quantile selection methods. The historical data methods have been used worldwide, while the precision of these methods strongly relies on the number and the independences of observations.

Unlike directly making use of the observations by historical data analysis methods, inferential models try to extrapolate the population of nutrients and chlorophyll a from the sample values. Regression models like the morphoedaphic index (MEI) methods, paleolimnological reconstruction approaches, and stressor-response models are included in this approach. MEI methods apply the regression relationships between TP and total ionic concentration and this method has been applied to estimate the TP reference conditions of lakes in Europe [7,12]. Subsequently, the improved MEI method for shallow lakes was used to work out the TP reference conditions of Taihu Lake in China [13]. The paleolimnological reconstruction approaches established regression models between TP and diatom chlorophyll concentration, which was considered a good estimation of diatom biomass [1,14,15,16]. Paleolimnological reconstruction approaches require data from a long time ago in sediment so they cannot be used for very shallow lakes where the sediment is affected by the wind wave [1,2]. The majority of stressor-response methods, which assign chlorophyll a as a response with TP, TN, and SD as the stressors [6], are also regression models. The reference conditions of lakes in several ecoregions in China have been estimated by these types of methods [17,18,19,20,21]. It should be noted that the reference conditions of chlorophyll a must be estimated before these stressor-response models are applied. Most of the change point detection methods performed by the U.S. EPA [6] are another kind of stressor-response approach. The change points were found in the stressor-response relationships and the nutrient reference conditions were worked out based on them. This type of method, which includes regression tree analysis (CART) [22], nonparametric change point analysis (nCPA) [23], and Bayesian hierarchical modeling (BHM) [24], was also developed to estimate the nutrient reference conditions of lakes in China.

Although inferential models have been widely used to work out reference conditions in lakes, the reliability and precision of these models, especially the regression models, rely on the independent distributed hypothesis of observations. This would lead to a biased estimation if the hypothesis was broken [25], so it is difficult to apply for time series or “cluster” observations.

The block generally extreme values (GEV) [26] and peak over threshold (POT) [27] theories was also used to infer the reference conditions of Taihu Lake. The two methods overcome the above faults by ensuring the independence of the observations as much as possible [28], with the cost that only a small number of observations can be used effectively. Only one observation per year can be applied in the block GEV method, while the POT method can utilize slightly more data. This causes a huge waste of data of which acquisition is difficult. Moreover, poor utilization of data leads to low precision for the estimation of nutrient reference conditions.

Based on the combination of Markov chain and extreme theories, a new method for estimating the nutrient reference conditions was proposed, and the reference values of TN, TP, and chlorophyll a in Taihu Lake were discussed and determined by the proposed method. This showed that the observations can be used more effectively and the precision of the reference values was higher than the results of GEV and POT.

## 2. Research Region and Data Sources

Taihu Lake lies in the south east of China, and is the third largest fresh water lake with an area of approximately 2445 km^2^. The lake is very shallow and the mean depth of Taihu Lake is only about 1.9 m. Around the lake, are the most developed cities of the country. Suzhou, Hangzhou, and Wuxi, which are located around the lake, had a GDP of more than 1 trillion dollars last year. Shanghai, the wealthiest and most dazzling city of China, is also close to the Taihu Lake. There is a population of a hundred million living around the lake and some of their water supply relies on it.

The water quality of Taihu Lake has seriously deteriorated over the last forty years. In the spring and summer, algae blooms occur frequently in the lake [29]. Although the government has spent a lot of resources to improve the water quality of Taihu Lake, it has not been effective. For the importance of the water quality of the lake, the estimation of its nutrient levels has attracted much attention.

**Figure 1:**
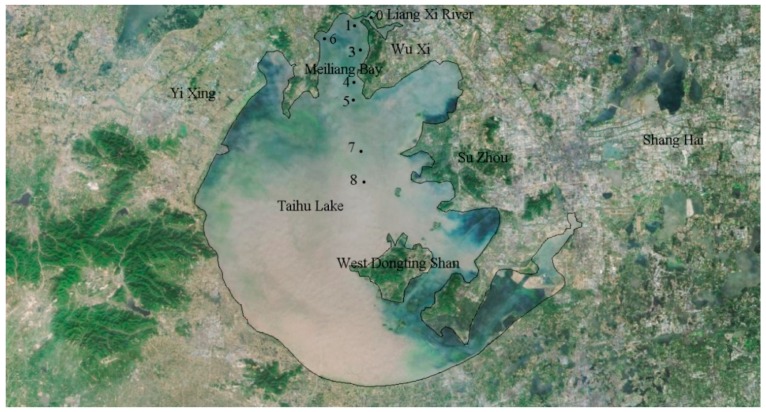
Locations of the sites in Taihu Lake.

The data used in the paper were obtained from the Chinese Ecosystem Research Network (CERN) who provided the TN, TP, and chlorophyll a observations from eight monitoring sites. Figure 1 shows the site locations [30]. Note that there is no site 2 due to historical reasons.

The observations of different sites at the same time can be considered as independent. The years of observations were from January 1995 to December 2006. Observations were conducted once a month and the experimental method was introduced in the reference. There were no missing data and 1152 available data points.

## 3. Methods

The Extreme–Markov method was developed from the Pareto distribution theory, which is also the basis of the POT method [28]. In detail, the method was based on three major assumptions: (1) The marginal distribution of observations in Taihu Lake was the generalized Pareto distribution (GPD); (2) The joint distribution of observations and next observations was a logistic function; (3) The observed data had the Markov property. The first assumption was confirmed in a previous study [27]. The second and third assumptions are checked in the results section.

The GPD theory shows that the observation should follow Equation (1) when it is higher than threshold value u. It can be obtained, like the POT method, by mean residual life plots [31].

(1) $$P(x_{t}-u<y|x_{t}>u)\to H(y)$$

H(y) is the GPD of y. It means that:

(2) $$H(y)=1-\left[1+\xi \frac{\left(y-u\right)}{\sigma }\right]{}^{-1/\xi },y>u$$

where $x_{t}$ is the observation data at t time. ξ and σ are the parameters of GPD. The two parameters can be worked out by the maximum likelihood method if the observations are independent. Then, the transformations

(3) $${\tilde{x}}_{t}=-\left(\ln\{1-\left[1+\xi \frac{\left(x_{t}-u\right)}{\sigma }\right]{}^{-1/\xi }\}\right){}^{-1}$$

induce a variable ${\tilde{x}}_{t}$ whose distribution is standard Fréchet for $x_{t}>u$.

According to the theory of multivariate extreme theory, the joint distributions function of ${\tilde{x}}_{t}$ and ${\tilde{x}}_{t-1}$ should be

(4) $$F({\tilde{x}}_{t},{\tilde{x}}_{t-1})=\exp[-V({\tilde{x}}_{t},{\tilde{x}}_{t-1})]$$

The function $V({\tilde{x}}_{t},{\tilde{x}}_{t-1})$ has to satisfy a set of conditions and the most commonly chosen is the logistic function [28,32], which says

(5) $$F(x_{t},x_{t-1})=F({\tilde{x}}_{t},{\tilde{x}}_{t-1})=\exp[-{\left({\tilde{x}}_{t}^{-1/\alpha }+{\tilde{x}}_{t-1}^{-1/\alpha }\right)}^{\alpha }]$$

The α is a dependent coefficient and α=0 means that $x_{t}$ and $x_{t-1}$ are independent, while α=1 means that $x_{t}$ is determined by $x_{t-1}$. The joint density function of $x_{t}$ and $x_{t-1}$ is

(6) $$f(\xi ,\sigma ,\alpha ;x_{t},x_{t-1})=\left\{ \begin{array}{ll} \frac{\partial^{2}F}{\partial x_{t}\partial x_{t-1}}{|}_{(x_{t},x_{t-1}),} & x_{t},x_{t-1}>u\\ \frac{\partial F}{\partial x_{t}}{|}_{(x_{t},u),} & x_{t}>u,x_{t-1}<u\\ \frac{\partial F}{\partial x_{t-1}}{|}_{(u,x_{t-1}),} & x_{t}<u,x_{t-1}>u\\ F(u,u) & x_{t},x_{t-1}<u \end{array}\right.$$

In the Extreme–Markov method, it is assumed that the observed data have Markov properties. It means that the probability density function of data $f(x_{t})$ obeys the following equation

(7) $$f(x_{t}|x_{t-1},x_{t-2},\cdots ,x_{1})=f(x_{t}|x_{t-1})$$

So, the likelihood function should be

(8) $$L(x_{1},\cdots ,x_{n};\xi ,\sigma ,\alpha )=h(x_{1},\xi ,\sigma )\prod_{t=1}^{n-1}\frac{f(\xi ,\sigma ,\alpha ;x_{t},x_{t-1})}{h(\xi ,\sigma ;x_{t})}$$

The $h(\xi ,\sigma ;x_{t})$ is the density function of GPD. The parameters in Equation (8) can be estimated by the maximum likelihood method, which must be solved by numerical technology in this condition. After that, the quantiles and their confidence interval can be worked out by the bootstrap method. Usually, the Extreme model deals with the maximum data so that the observations should take the opposite in order to work out the reference conditions [28].

## 4. Results

Table 1 shows the characteristics of the observations at the eight sites in Taihu Lake. The mean and maximum value of TN, TP, and chlorophyll a were very high at these sites. This implies that eutrophication had been a great problem for Taihu Lake for these years. The kurtosis and skew of observations showed the distributions of the values deviated seriously from the Gaussian to higher values. All of these statistics indicated that the water quality of Taihu Lake was terrible and had been seriously affected by human activities, so its nutrients and chlorophyll a reference conditions have to be established carefully from these observations.

**Figure 2:**
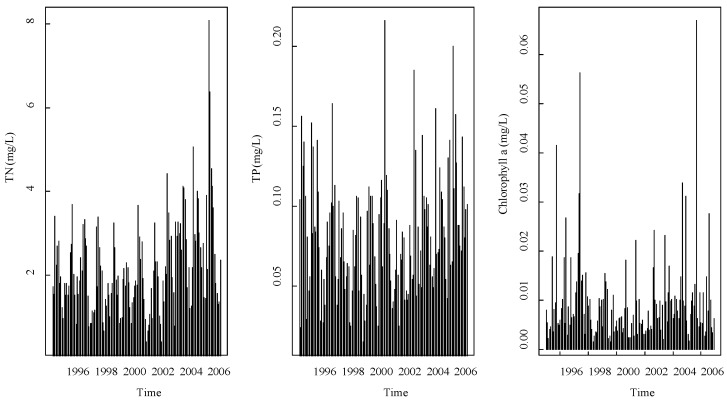
Time series of total nitrogen (TN), total phosphorus (TP), and chlorophyll a.

Figure 2 shows the TN, TP, and chlorophyll a time series of site 7 while the observations of the other sites are not shown since they were similar to this one.

Figure 2 implies that there was a “cluster” phenomenon in both the maximum and minimum values. This means that high observations were followed by high values while low values came after low concentration. This may be because the observations of TN, TP, and algae growth were strongly influenced by the nature condition, which usually appears as a cluster.

**Figure 3:**
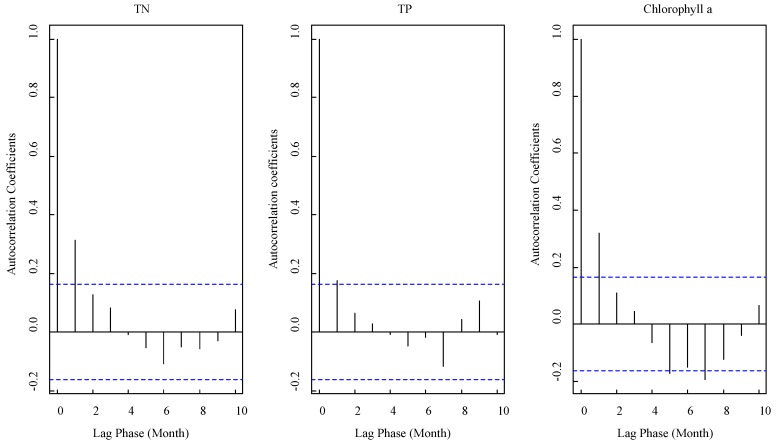
The autoregressive coefficients of TN, TP, and chlorophyll a. The solid lines are the results of the coefficients, the dotted lines show the boundaries of the 95% confidence interval.

Figure 3 shows the autoregressive coefficients of the TN, TP, and chlorophyll a observations at site 7. Unsurprisingly, the results indicated that there was a dependence between the observations and the values following it. The necessary actions to apply the Markov model were performed to deal with this dependence. Furthermore, in most figures only the lag 1 coefficients were significant under 95% confidence because the nature environment, which affects water quality, using temperature and flow rate as examples, showed obvious differences after two or three months in the Taihu Lake region. This also implied that the first order Markov model may be suitable to manage the dependence in these observations.

The cluster and dependence of the observations’ time series indicated that the quantile selection method-based independence hypothesis, such as the frequency analysis method, could not be applied. Most observations must also be given up to keep the data independent in the block GEV method [26] and the POT model [27]. In fact, the waste of data made almost all the methods, which were based on the time series observations, show a fall in the precision of the results [33,34].

**Figure 4:**
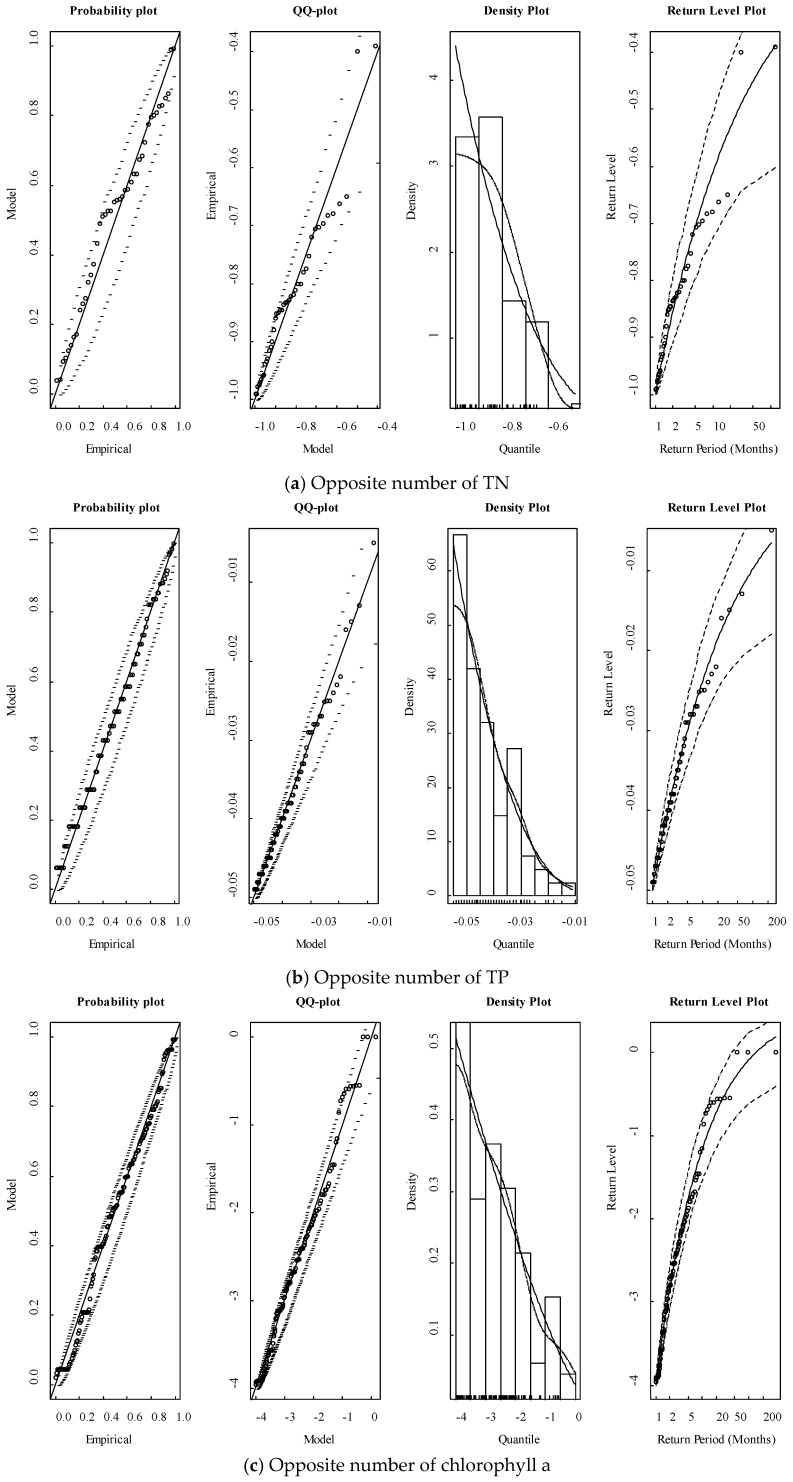
Diagnostic plots of the Markov–Extreme model on the opposite number of TN, TP, and chlorophyll a. The solid lines are the model results, dotted lines show the boundaries of the 95% confidence interval, and the points are the opposite number of observations higher than the thresholds.

As the data used in this paper were the same as in the reference paper, the thresholds that were u in Equation (1) of the opposite number of TN, TP, and chlorophyll a could also be the same as in this paper, which were −1.0 mg/L, −0.05 mg/L, and −4 μg/L [27]. The results showed that these thresholds were suitable for the extreme model. Figure 4 showed the results of the Extreme–Markov model diagnostic on the opposite number of TN, TP, and chlorophyll a.

Both the probability, quantiles–quantiles (QQ), and return level plots implied that all of the opposite number of observations higher than the threshold were included in the 95% confidence interval boundaries of the Extreme–Markov models. This implied that the observations of TN, TP, and chlorophyll a fitted the model well.

## 5. Discussion

Table 2 shows the comparisons of the POT and Extreme–Markov theories, where the model forms were similar to each other, and were estimated by the same data with the same thresholds.

The number of observations was almost twice that of the POT model, which means that the data could be more effectively used in the Extreme–Markov model for estimating the reference condition. The standard errors (se) of the model parameters were only about 50% smaller than that of the POT model. This suggests that the precision of the Extreme–Markov models was significantly higher than the POT models. Furthermore, the α in the model was less than 1, meaning that there was significant dependence between the effective observations, especially in the chlorophyll a model where α<0.5, so the dependence must be considered when the reference conditions models are built by time series data in Taihu Lake.

Corresponding to the quantiles selection method, a certain quantile value would be specified as the reference conditions. Considering the serious eutrophication state of Taihu Lake, Zheng [11] selected 5% quantiles as the reference values using the frequency analysis method. The same quantiles had also been recommended as the reference values of Taihu Lake by the seasonal decomposition model [33]. Chen et al. [10] also inferred the reference conditions of Chaohu Lake, whose state was very similar to Taihu Lake when using 5% quantiles.

Although 5% quantiles have been selected for several methods and studies in Taihu Lake, 25% quantiles, which have also been applied by the U.S. EPA [1,2] for the reference lakes method, have been recommended in extreme models [26,27] as the extreme model used the better data of Taihu Lake. The 25% quantiles were applied in this paper as the Extreme–Markov method is also one type of extreme model.

Table 3 shows the reference conditions and their 95% confidence intervals of Taihu Lake inferred by the Extreme–Markov approach, the POT model, and the GEV model.

Table 4 gives the results of the reference conditions in Taihu Lake by other methods for comparison. All of them only provided the point estimation except for the seasonal decomposition model [33], of which the 95% confidence intervals are also shown.

As shown in the tables, the TN and TP reference values estimated by the proposed method were very similar to the results worked out by the POT, GEV, frequency analysis, and seasonal decomposition methods. The Chinese Academy of Sciences conducted a large scale investigation into the water quality of Taihu Lake in the 1960s [35] and the results showed that the observations of TP were about 0.01–0.05 mg/L. The median was 0.030 mg/L. Considering that the water pollution in Taihu Lake grew after the 1980s, the median of TP values could be used as a reference point reflecting the water quality less impacted by humans. It also supports the fact that the reference conditions of TP estimated by the Extreme–Markov models are appropriate. The chlorophyll a reference values were a little lower than those of the frequency analysis method, however, this was unsurprising. The data used in the reference [11] was in the 1990s and algae bloom occurred frequently during this period in Taihu Lake. This would certainly have caused the chlorophyll a observations to be elevated abnormally so that the reference condition worked out from these data would be significantly high. Generally speaking, considering the resemblance between the reference values calculated by the proposed model and previous research using many different methods, the reference conditions obtained by the Extreme–Markov model were reliable.

Compared with the results of the POT using the same data and threshold values, the confidence interval calculated by the proposed method was reduced by 36.3%, 33.3%, and 9.3%, respectively. For the results of the GEV methods, the reduced ratios were 46.1%, 86.7%, and 32.6% and for the seasonal decomposition method, it was reduced by 46.7%, 90.5%, and 13.9%. These results imply that the Extreme–Markov theory performed with a remarkably higher precision than the POT, GEV, and seasonal decomposition method.

## 6. Conclusions

The nutrient reference conditions of lakes are very important for water quality protection. Since there are dependent and “cluster” in time series observations, historical data analysis such as the frequency analysis method is difficult to apply with time series data. The POT and GEV methods included in inferential models can be used, but waste a lot of data, leading to low precision in the estimation of nutrient reference conditions.

A new reference condition estimation method based on extreme and Markov chain theories was proposed. In this method, the GPD was the marginal distributions of the opposite number of TN, TP, and chlorophyll a observations. The dependence among the observations was modeled by logistic function, and then the likelihood function of the model was obtained on the basis of the Markov chain hypothesis. More observations with serious “cluster” and dependences in time series could be applied to estimate the reference conditions by this method. The proposed approach was employed to work out the reference conditions of Taihu Lake. The results showed that the method was reliable and that there was notable dependence between the effective observations of TN, TP, and chlorophyll a. The 25% quantiles of the proposed models were recommended as the reference conditions of TN, TP, and chlorophyll a in Taihu Lake. The corresponding values were 0.69 mg/L, 0.029 mg/L, and 1.89 μg/L, and the 95% confidence intervals were 0.62–0.76 mg/L, 0.028–0.030 mg/L, and 1.55–2.23 μg/L, respectively. It also showed that the Extreme–Markov method could reduce the 95% confidence intervals significantly in comparison to the results of the POT, GEV, and seasonal decomposition approach. This means that this method remarkably improved the precision of the reference values. This provides a useful exploration for the estimation of lake reference conditions and can be extended to the study and management of other lakes.

## Figures and Tables

**Table 1:** The statistics of the observations in Taihu Lake.

	Total Nitrogen (mg/L)	Total Phosphorus (mg/L)	Chlorophyll a (μg/L)
Mean Value	4.01	0.16	24.78
Standard Deviation	2.81	0.14	34.57
Kurtosis	2.85	49.19	53.28
Skew	1.54	5.06	5.40
Minimum Value	0.39	0.00	0.00
Maximum Value	21.93	2.13	521.73

**Table 2:** Comparison of the parameters between the peak over threshold (POT) and Markov–Extreme model results.

		POT Model [27]			Extreme–Markov Model		
Variables	Effective Observations	*σ*(se)	*ξ*(se)	Effective Observations	*σ*(se)	*ξ*(se)	*α*(se)
TN	77	0.34(0.093)	−0.48(0.21)	129	0.23(0.044)	−0.27(0.12)	0.78(0.046)
TP	97	0.028(0.002)	−0.58(0.051)	162	0.015(0.001)	−0.17(0.033)	0.88(0.032)
Chlorophyll a	127	3.27(0.53)	−0.82(0.15)	278	1.94(0.22)	−0.43(0.08)	0.17(0.004)

**Table 3:** The estimation of the reference conditions and 95% confidence intervals by the POT, block generally extreme values (GEV) and Extreme-Markov models.

Variables	POT Model [27]		GEV Model [26]		Extreme–Markov Model	
	25% Quantiles	95% Confidences	25% Quantiles	95% Confidences	25% Quantiles	95% Confidences
TN (mg/L)	0.66	0.55–0.77	0.71	0.58–0.84	0.69	0.62–0.76
TP (mg/L)	0.023	0.022–0.025	0.025	0.018–0.033	0.029	0.028–0.030
Chlorophyll a (μg/L)	1.27	0.84–1.70	1.81	1.32–2.33	1.89	1.55–2.23

**Table 4:** The recommended reference conditions by previous research.

Variables	Methods	Reference Values (95% Confidence Intervals)
TN (mg/L)	Frequency Analysis [11]	0.60
	Seasonal Decomposition [33]	0.78 (0.53–0.83)
TP (mg/L)	Frequency Analysis [11], MEI [13]	0.030
	Seasonal Decomposition [33]	0.030 (0.025–0.046)
Chlorophyll a (μg/L)	Frequency Analysis [11]	4
	Seasonal Decomposition [33]	2.63 (1.86–2.65)
